# The Association Between Childhood Impaired Motor Development and Adolescent Psychotic Experiences

**DOI:** 10.1002/dev.70049

**Published:** 2025-05-14

**Authors:** P. J. Hamers, D. C. Bouter, S. Dieleman, W. J. G. Hoogendijk, N. H. Grootendorst ‐ van Mil

**Affiliations:** ^1^ Erasmus MC University Medical Center, Rotterdam University Medical Center Rotterdam Rotterdam Netherlands; ^2^ Epidemiological and Social Psychiatric Erasmus MC University Medical Center, Department of Psychiatry University Medical Center Rotterdam Rotterdam Netherlands

**Keywords:** adolescent, delusions, hallucinations, high‐risk, motor development, population based, psychotic experiences

## Abstract

Increasing evidence indicates that psychosis spectrum disorders are neurodevelopmental disorders linked to early life. Motor impairments are proposed as a key early marker of risk for psychosis spectrum disorders. Here, we explored the association between childhood impaired motor development and psychotic experiences (PE) in adolescents. Participants were 658 adolescents from a cohort oversampled on their self‐reported emotional and behavioral problems. Parents reported retrospectively on childhood motor development, including onset of walking, aptitude in ballgames, balance, and smoothness of movement. Adolescents completed the Prodromal Questionnaire (PQ‐16) to assess PE at two time points (mean age 14.73 at first measurement and 17.78 at follow‐up). Multiple linear regression analyses were performed to assess associations between childhood impaired motor development and adolescent PE. Childhood impaired motor development was associated with higher levels of adolescent PE (*β* = 0.23, 95% CI 0.08; 0.38) at age 17, but not at age 15. In addition, motor impairments were associated with an increase in PE between the two time points. This association was especially apparent in hallucinatory experiences (*β* = 0.26, 95% CI 0.13; 0.39), but not in delusional experiences. Childhood impaired motor development may signal an increased risk for adolescent PE, emphasizing the need for precise assessment tools and further research into these associations. This study supports the notion of psychosis spectrum disorders as neurodevelopmental in nature and highlights the role of early risk markers in identifying these disorders.

## 1 Introduction

The recognition of childhood impaired motor development as an indicator of increased psychosis risk has gained substantial attention in recent research (Whitty et al. 2009; Walther and Strik 2012; Peralta and Cuesta 2017; T. Damme et al. 2015; Mittal and Wakschlag 2017; Karcher et al. 2023; K. Damme et al. 2022; Burton et al. 2023). Studies have consistently linked motor difficulties with the entire spectrum of psychosis disorders, including those individuals who are considered at risk due to affected family members. This underscores the significance of motor abnormalities in unraveling the origins of psychosis spectrum disorders. Childhood motor impairment includes a range of deficits in motor performance that become evident during the developmental years, encompassing challenges from gross motor difficulties, such as clumsiness or awkward physical movements, to subtler issues like impairments in fine motor skills, abnormalities in motor speed, coordination deficits, and unusual movements or gestures (Vaivre‐Douret et al. 2016).

A recent study by Burton et al. (2023) revealed that in children at high familial risk for schizophrenia, there was a higher likelihood of impaired motor development, as determined through a comprehensive test battery evaluated by a clinical rater. Notably, impaired motor development was associated with a higher frequency of psychotic experiences (PE, i.e., hallucinations or delusions), a trend that was especially strong in the high‐risk group but was also observed in the general population without specific familial risk factors. The authors suggest that motor developmental impairments could reflect both genetic vulnerability and environmental exposure, as well as the interaction of the two. They speculate that environmental factors such as the level of motor stimulation and sports activities might impact the development of motor function.

The pathophysiological role of motor development and impairment in the etiology of psychosis spectrum disorders is still largely unknown. Several explanations have been put forward, including vulnerability of the striatal system, showing impaired motor development early and psychopathological symptoms later (O. Howes et al. 2011), underlying frontal–striatal circuit pathology (Dean and Mittal 2015), or pathology of cerebellar substructures and connectivity (Bernard et al. 2014; Bernard and Mittal 2015; Dean et al. 2016). However, none of these proposed explanations have been conclusively established.

The association between PE and an increased incidence of later development of psychosis spectrum disorders is well established, with a meta‐analysis finding a fourfold increased risk compared to the general population (Kirli et al. 2019; Healy et al. 2019). PE are also associated with an increased risk of several other psychopathologies, such as mood disorders, anxiety disorders, substance‐use disorders, and behavioral disorders (Karcher et al. 2023; Kirli et al. 2019; Rejek and Misiak 2023; Steenkamp et al. 2023; Palstra et al. 2023). Similarly, to how PE serve as transdiagnostic indicators for a range of psychopathologies, motor abnormalities also extend their significance beyond marking susceptibility to psychosis. Previous research highlighted that such abnormalities are indicative of a shared vulnerability for PE, depressive symptoms, particularly when there is a concurrent familial history of PE and depression (K. Damme et al. 2022). Further research supports the association between developmental motor delays and familial risk for depression or psychosis, with psychomotor retardation specifically linked to depression risk (O. Howes et al. 2011). These findings emphasize the significance of motor abnormalities as early indicators of various mental health conditions (Van Harten and Pieters 2022). Moreover, emerging evidence suggests a potential link between impaired motor development and bipolar disorder, expanding the scope of psychopathological conditions associated with motoric indicators (K. Damme et al. 2022; Walther and Mittal 2022; Sugranyes et al. 2017).

Even though PE are a well‐studied subject and have, during recent years, also been studied in the context of motor abnormalities and impaired motor development, almost all of these studies are done in populations selected on familial‐risk, thus focusing more on inheritance patterns (Whitty et al. 2009; Walther and Strik 2012; Peralta and Cuesta 2017; T. Damme et al. 2015; Mittal and Wakschlag 2017; Karcher et al. 2023; K. Damme et al. 2022; Burton et al. 2023). In addition, currently there are no studies that distinguish between different types of PE, making it impossible to distinguish specific etiological trajectories.

In the present study, we examine the association between childhood impaired motor development and PE in late adolescence in a population‐based at‐risk cohort. Specifically, we focused on delayed motor milestones and underperformance in motor skill and coordination relative to peers. In regard to PE, we examined the associations for both the total score and the subdomains hallucinatory and delusional experiences at two separate time points. In addition, we analyzed changes in these three scores over time by assessing their delta scores. Considering the established connection between impaired motor development and affective disorders, our study aims to ascertain whether these associations remain significant after adjusting for concurrent emotional and behavioral issues.

## 2 Materials and Methods

### 2.1 Participants

This study was conducted within the iBerry (Investigating Behavioral and Emotional Risk in Rotterdam Youth) study, a prospective cohort study in the Netherlands that aims to investigate the development of subclinical symptoms into psychiatric disorders. The iBerry cohort consists of adolescents that filled out the Strengths and Difficulties Questionnaire Youth (SDQ‐Y) as part of a health survey conducted by the CJG Rijnmond (Center for Youth and Family of the greater Rotterdam area). The 25‐item SDQ‐Y is one of the most widely used instruments for assessing self‐reported emotional and behavioral symptoms in children and adolescents (Goodman et al. 2003; Muris et al. 2003; van Widenfelt et al. 2003).

Of the 16,736 adolescents who filled out the SDQ‐Y, all high‐risk individuals (top 15% highest scores) were invited to participate, along with a random selection of lower‐risk adolescents (lowest 85% scores). High‐risk adolescents were oversampled in a 2.5:1 ratio to create sufficient power to study less‐common outcomes. The cohort has been described in further detail elsewhere (Grootendorst‐van Mil et al. 2021). At baseline, from 2015 to 2019, 1022 adolescents participated in the study (54% response rate, mean age 15.0 years). A total of 661 parents filled out an additional questionnaire regarding their child's development 9 months after the baseline appointment. At the first follow‐up measurement, 807 adolescents participated (78.9% response rate, mean age 18.1 years) (van Widenfelt et al. 2003). For each visit, written informed consent was obtained. Information was acquired through interviews, questionnaires, various tasks, and physical measurements.

Participants received a small monetary compensation for their participation. The iBerry study protocols for both baseline and follow‐up measurements were approved by the Erasmus Medical Center's Medical Ethics Review Committee (MEC‐2015‐007 and MEC‐2018‐1472).

### 2.2 Measures

#### 2.2.1 Childhood Impaired Motor Development

Childhood impaired motor development was assessed using questions on motor impairment from a parent‐reported questionnaire, similar to the one employed in the TRAILS study and adapted from validated assessment tools: the Child Development Inventory (CDI) and the Movement Assessment Battery for Children (MABC) (Henderson et al. 2007). This compiled questionnaire focuses on the early life stages of the child, from birth to toddler age and was designed to limit the burden on parents by being concise and easy to complete. In the questions used for this study, specifically, parents were inquired about the age at which their child was able to walk 3–5 steps unassisted. The response options included: “don't know/no answer,” “very early (10 months or earlier),” “early (11–12 months),” “average (13–17 months),” “late (18–23 months),” and “very late (24 months or later).”

Balance and general fine motor skills were assessed with three questions: was your child skillful in ballgames? Was your child able to maintain his/her balance (standing on one leg, for example)? And: did your child move smoothly? Parents assessed their child as either “below average,” “average,” or “above average.” For analyses, a parent‐reported “late or very late” or “below average”‐score was regarded as impaired motor development. The four questions were analyzed independently, as well as in two combined scores: a continuous sum score of the four items and a dichotomous score of impaired motor development on at least one of the four items.

#### 2.2.2 Psychotic Experiences

To measure PE, we used the Prodromal Questionnaire 16 (PQ‐16). This self‐report questionnaire consists of 16 items that are rated true or false. The PQ‐16 has good psychometric properties; a high score on this questionnaire identifies adolescents at risk of psychosis (Ising et al. 2012). In subsequent analyses, two subscales of the PQ‐16 were examined, hallucinatory experiences and delusional experiences, along with a calculated score representing the difference in PE between the two conducted measurements.

### 2.3 Sociodemographic and Clinical Characteristics

Age and education level of the adolescent and family income were obtained with general questionnaires. Education level was reported by the adolescent and divided into three categories: low education level represents (pre)vocational education, medium represents higher (pre)vocational education, and high represents (pre‐)university education. Net monthly family income was reported by the accompanying parent and divided into the following categories: <€1600, €1600–€2399, €2400–€4400, or > €4400. The educational level of the reporting parent was collected and categorized into five groups: primary school, secondary school/vocational training, HBO (Higher Professional Education), WO (university education), and Other. Urbanicity was categorized as rural, suburban or urban, based on the number of addresses per km^2^ surrounding the adolescents’ home address. These categories were defined as < 1000, 1000–1500 and > 1500 addresses/km^2^, respectively, following definitions by Statistics Netherlands (Statistics Netherlands 2007). The Youth Self‐Report (YSR) was utilized to evaluate emotional and behavioral problems. The YSR is a comprehensive questionnaire designed for adolescents to self‐assess their emotional and behavioral issues. It covers a wide range of aspects, including internalizing problems (such as anxiety and depression), externalizing problems (like aggressive and rule‐breaking behaviors), and other specific syndromes (Achenbach 2001).

### 2.4 Statistical Analysis

The associations between childhood impaired motor development and adolescent PE were assessed with multiple linear regression analyses. All analyses were performed in IBM SPSS Statistics 28 (IBM Corp. 2021). Because the PE measurements displayed skewed distribution, they were transformed using a square root function to approximate normality. All analyses were adjusted for age and sex.

Sensitivity analyses were conducted to examine the associations between impaired motor development and PE after additional adjustment for co‐occurring emotional and behavioral problems. We reasoned that, since we are examining the association between two neurodevelopmental/psychiatric outcomes within the same individual, the risk of confounding would be minimal. Therefore, additional adjustments for socioeconomic or clinical characteristics were not included. However, to test for potential influences of socioeconomic background on parent‐reported measures, analyses were additionally adjusted for the educational level of the reporting parent.

For non‐response analyses, we performed a *t*‐test for continuous variables and Chi‐square tests for categorical variables to compare adolescents included in the analyses with those not included based on missing data.

## 3 Results

### 3.1 Participant Characteristics

The participant characteristics and descriptive statistics are provided in Table 1. Childhood impaired motor development and PE were available for 658 (64.4% of total *n* = 1022) adolescents at the first measurement and 584 adolescents (72.5% of total *n* = 807) at the second measurement. The mean age of adolescents was 14.73 years at the first measurement and 17.87 years at the second measurement.

**TABLE 1:** Sociodemographic characteristics and descriptive statistics for measures of study sample.

Variable	T0 (*n* = 658)	T1 (*n* = 584)
Age, years, mean (SD)	14.73 (0.76)	17.87 (0.76)
Sex, *n* (%) Male Female	316 (48.0) 342 (52.0)	269 (46.1) 315 (53.9)
Adolescent education level, *n* (%) Low Medium High Mixed levels Missing	292 (44.4) 151 (23.0) 153 (23.3) 57 (8.7) 5 (0.8)	287 (49.1) 167 (28.6) 124 (21.2) 6 (1.1)
Urbanicity, *n* (%) Rural Suburban Urban	137 (20.8) 134 (20.4) 387 (58.8)	128 (22.0) 110 (18.8) 346 (59.2)
Household monthly income, *n* (%) < €1600 €1600–€2399 €2400–€4400 > €4400 Missing	52 (7.9) 97 (14.7) 331 (50.3) 149 (22.7) 29 (4.4)	25 (4.3) 76 (13.0) 263 (45.0) 214 (36.6) 6 (1.1)
Parental education level, *n* (%) Low Medium High Missing	373 (56.7) 147 (22.3) 99 (15.1) 39 (5.9)	329 (56.3) 155 (26.6) 96 (16.4) 4 (0.7)
Emotional and behavioral problems, score, median (IQR)	38 (24)	41 (30)
Onset of walking, *n* (%) Very early (< 10 months) Early (11–12 months) Average (13–17 months) Late (18–23 months) Very late (> 24 months) Missing	57 (8.7) 178 (27.0) 360 (54.7) 46 (7.0) 3 (0.5) 14 (2.1)	51 (8.7) 151 (25.9) 323 (55.3) 44 (7.5) 3 (0.5) 12 (2.1)
Aptitude in ballgames, *n* (%) Below average Average Above average Missing	147 (22.3) 381 (57.9) 129 (19.6) 1 (0.2)	138 (23.6) 330 (56.5) 115 (19.7) 1 (0.2)
Balance (i.e., standing on one leg), *n* (%) Below average Average Above average	93 (14.1) 416 (63.2) 149 (22.7)	80 (13.7) 375 (64.2) 129 (22.1)
Smoothness of movement, *n* (%) Below average Average Above average Missing	81 (12.3) 394 (59.9) 182 (27.7) 1 (0.1)	70 (12.0) 356 (61.0) 158 (27.1) 0 (0.0)

Abbreviations: IQR = Interquartile Range; SD = Standard Deviation.

At the first measurement of PE, a total of 209 (31.8%) adolescents were reported as displaying at least one sign of childhood impaired motor development; during the second measurement of PE, this number was 189 (32.4%). The average total sum score for the measures of impaired motor development was 0.55 (SD = 0.97) for the first measurement and 0.57 (SD = 0.98) for the second measurement, with a total range from 0 to 4. In both measurements. The most common measures of impaired motor development were below‐average aptitude in ballgames (22.3% and 23.6%) below‐average balance (14.1% and 13.7%), and below‐average smoothness of movement (12.3% and 12.0%). Late onset of walking was reported for 7.5% and 8% of the adolescents at the two measurements, respectively.

### 3.2 Impaired Motor Development and PE

Table 2 shows the results of the linear regression analyses of the associations between the various measures of childhood impaired motor development and adolescent PE at two separate time points, as well as the change in PE scores (delta scores) between these measurements. At the first measurement (mean age 15), there was an association between delayed onset of walking and total PE. At the second measurement (mean age 18), there was an association between below‐average aptitude in ballgames and total and specifically hallucinatory experiences. An association was also found between below‐average balance and hallucinatory experiences. When examining the combined motor development scores, we found that the continuous combined score of all items was significantly associated with total PE and hallucinatory experiences at the second measurement. The presence of at least one sign of childhood impaired motor development displayed significant associations with total PE at both measurements and specifically with hallucinatory experiences at the second measurement. However, no associations were found with delusions independently at either measurement. Lastly, a below‐average aptitude in ballgames was also associated with the increase in hallucinatory experiences between the first and second measurements.

**TABLE 2:** Associations between childhood impaired motor development and adolescent psychotic experiences.

Impaired motor development	Psychotic experiences					
	T0 – mean age = 15.0 years—*n* = 658					
	Total psychotic experiences		Hallucinatory experiences		Delusional experiences	
	*β* (95% CI)	*p* value	*β* (95% CI)	*p* value	*β* (95% CI)	*p*‐ value
Onset of walking	**0.29 (0.02; 0.55)**	**0.04**	0.20 (−0.05; 0.44)	0.12	0.18 (−0.02; 0.37)	0.08
Aptitude in ballgames	0.09 (−0.08; 0.26)	0.29	0.06 (−0.10; 0.21)	0.47	0.03 (−0.10; 0.15)	0.67
Balance	0.13 (−0.08; 0.33)	0.23	0.16 (−0.03; 0.35)	0.09	0.00 (−0.15; 0.15)	1.00
Smoothness of movement	0.08 (−0.14; 0.29)	0.49	0.11 (−0.09;0.31)	0.28	−0.04 (−0.20; 0.11)	0.60
Sum scores:						
Continuous	0.06 (−0.02; 0.13)	0.15	0.05 (−0.02; 0.12)	0.14	0.01 (−0.04; 0.06)	0.70
Above cutoff (≥ 1 impairment)	**0.15 (0.00; 0.31)**	**0.05**	0.10 (−0.04; 0.24)	0.16	0.05 (−0.06; 0.16)	0.35

	T1 – mean age = 18.1 years—*n* = 584					
	Total psychotic experiences		Hallucinatory experiences		Delusional experiences	
	*β* (95% CI)	*p*‐value	*β* (95% CI)	*p* value	*β* (95% CI)	*p* value
Onset of walking	0.08 (−0.19; 0.34)	0.57	0.01 (−0.22; 0.25)	0.91	0.12 (−0.08; 0.32)	0.24
Aptitude in ballgames	**0.23 (0.06; 0.39)**	**0.007**	**0.28 (0.13; 0.42)**	**< 0.001**	0.02 (−0.11; 0.14)	0.80
Balance	0.16 (−0.05; 0.36)	0.14	**0.29 (0.10; 0.47)**	**0.002**	−0.05 (−0.21; 0.11)	0.52
Smoothness of movement	0.10 (−0.12; 0.32)	0.36	0.18 (−0.01; 0.38)	0.07	−0.06 (−0.23; 0.11)	0.46
Sum scores:
Continuous	**0.08 (0.01; 0.16)**	**0.03**	**0.11 (0.05; 0.18)**	**0.001**	0.00 (−0.06; 0.05)	0.88
Above cutoff (≥ 1 impairment)	**0.23 (0.08; 0.38)**	**0.002**	**0.26 (0.13; 0.39)**	**< 0.001**	0.07 (−0.04; 0.19)	0.23

	Difference between T0 and T1–*n* = 582					
	Total psychotic experiences		Hallucinatory experiences		Delusional experiences	
	*β* (95% CI)	*p* value	*β* (95% CI)	*p* value	*β* (95% CI)	*p* value
Onset of walking	−0.10 (−0.95; 0.76)	0.83	−0.13 (−0.66; 0.40)	0.63	0.10 (−0.27; 0.47)	0.60
Aptitude in ballgames	0.47 (−0.07; 1.01)	0.09	**0.44 (0.11; 0.78)**	**0.01**	−0.02 (−0.26; 0.21)	0.85
Balance	0.11 (−0.56; 0.79)	0.75	0.20 (−0.22; 0.61)	0.36	−0.12 (−0.41; 0.17)	0.41
Smoothness of movement	0.14 (−0.57; 0.86)	0.70	0.15 (−0.30; 0.60)	0.51	−0.04 (−0.35; 0.27)	0.79
Sum scores:						
Continuous	0.15 (−0.09; 0.39)	0.22	0.14 (−0.01; 0.29)	0.06	−0.01 (−0.11; 0.10)	0.87
Above cutoff (≥ 1 impairment)	0.32 (−0.17; 0.82)	0.20	0.27 (−0.04; 0.57)	0.09	0.07 (−0.15; 0.28)	0.55

*Note:* Results from linear regression models, adjusted for age and sex; psychotic experiences were assessed with the Prodromal Questionnaire 16.

### 3.3 Sensitivity Analyses

Non‐response analyses showed that non‐responders had lower education, family income, and, at 18 years, more urban residency. No differences were found in age, sex, or psychopathology scores (Table S1).

Additional adjustment for emotional and behavioral problems only slightly diminished the associations found between hallucinatory experiences and childhood impaired motor development at the second measurement. However, the associations between the PE total score and childhood impaired motor development at both measurements were no longer significant. Contrastingly, adjustment for emotional and behavioral problems resulted in somewhat stronger associations between childhood impaired motor development and the change in PE scores between both measurements. Aside from the association with below‐average aptitude in ball games, additional associations were observed between below‐average aptitude in ball games and the difference in total PE scores. The difference in hallucinatory experiences was associated with both the continuous combined score and the presence of at least one motor impairment (Table S2). Adjusting for the educational level of the reporting parent did not significantly alter the findings (results not shown).

## 4 Discussion

This study found an association between parent‐reported childhood impaired motor development and PE in adolescence. Impaired motor development relative to peers was associated with PE at age 18, but not at age 15. In line, motor impairments were linked to an increase in PE between the two time points. Specifically, below‐average aptitude in ball games and impaired balance were predictive of adolescent PE and hallucinatory experiences, rather than delusions alone. These results were specific to PE and were not explained by co‐occurring emotional and behavioral problems.

Our findings are in line with previous studies that have indicated an association between impaired motor development or other motor abnormalities and PE (Walther and Strik 2012; Peralta and Cuesta 2017; T. Damme et al. 2015; K. Damme et al. 2022; Walther and Mittal 2022). Our findings support the existing evidence, suggesting that early neurodevelopmental factors, such as impaired motor development, precede the risk of psychosis. Previous studies have shown that individuals with increasing or persistently high levels of PE are more frequently exposed to early life adversity, substance use, and other psychopathological factors (Mackie et al. 2011; Wigman et al. 2011; Thapar et al. 2012). Impaired motor development may not only act as a trigger for PE, but both impaired motor development and PE could result from early life adversity and other detrimental factors. In our study, we observed associations between impaired motor development and PE at age 18, but not at age 15, which aligns with the increase in PE observed between ages 15 and 18. The full impact of altered early neurodevelopment may become evident only later in adolescence, as (additional) environmental stressors exacerbate existing vulnerabilities.

Unlike previous studies, however, this study distinguished between hallucinatory and delusional experiences. Remarkably, we found no association between impaired motor development and delusional experiences. The reason this association is lacking could be multifold. First, the discrepancy in our findings regarding hallucinatory and delusional experiences underlines the notion of an etiological difference between the two, with hallucinatory experiences being more closely related to (causes of) the divergent neurodevelopment that plays a part in the occurrence of impaired motor development and/or the eventual onset of psychosis spectrum disorders. Previous studies support the notion that hallucinatory and delusional experiences are disparately associated with other psychosocial measures. Hallucinatory experiences were associated with an increased likelihood of depression and insufficient sleep, whereas delusional experiences were inconsistent in their associations (Hielscher et al. 2018). Similarly, others found that hallucinations were associated with higher distress, depression, and worse functioning (Armando et al. 2010), implying the clinical relevance of hallucinatory experiences. This notion is further substantiated by the findings of the current study, which are consistent with the idea that hallucinatory experiences are part of a group of factors associated with the development of psychosis spectrum disorders, possibly via aberrant neurodevelopment. However, the exact neurophysiological mechanisms behind this etiological trajectory remain largely unknown. Both impaired motor development and PE share overlapping cortical and subcortical networks. Most important are the cerebellum and basal ganglia, involved in motor control and dopaminergic modulation, and as a consequence dopaminergic circuits, essential for both movement and salience attribution (Andreasen and Pierson 2008; O. D. Howes and Kapur 2009). The current consensus in neuroscientific literature is that these neural pathways, when atypically developed, manifest first as impaired motor development and later as PE (Cannon et al. 2002). Research suggests that these pathways are implicated in both hallucinations and delusions (O. D. Howes and Kapur 2009; Allen et al. 2008; Coltheart 2010; Fletcher and Frith 2009). For instance, both phenomena have been associated with dopaminergic hyperactivity (Kapur 2003). Therefore, as of yet, no suitable neurophysiological explanation for the disparate association found in this study is available, only the aforementioned clinical disparities. Alternatively, difficulties with motor tasks can hinder social participation, potentially leading to isolation and increased stress, which are known risk factors for developing PE (De Roubaix et al. 2024). In regard to measures of impaired motor development, research suggests that aptitude in ballgames and balance are likely more sensitive markers of neurodevelopment than milestones like the age of onset of walking. These tasks require complex coordination, multisensory integration, and higher‐order system, including the cerebellum, motor cortex, and prefrontal regions (Schmahmann 2019). Thus, the associations found may reflect more advanced or integrative neural processes, potentially tied to cerebellar or dopaminergic dysfunction. These brain systems are not only central to motor control but also implicated in salience attribution and cognitive processes underlying hallucinations and delusions. While our study cannot confirm a specific mechanism, these findings may point toward distinct developmental trajectories or neural substrates that underlie PE. Further, motor difficulties may impact social engagement and stress sensitivity—factors also linked to psychosis risk. In support of this view, Filatova et al. (2017) conducted a meta‐analysis showing that delays in walking, sitting, and standing unsupported—milestones achieved later in infancy—were significantly associated with schizophrenia in adulthood. In contrast, earlier milestones such as holding the head up and grabbing objects were not predictive. The study by Filatova et al. (2017) highlights that walking, as one of the most complex motor milestones to emerge, may be a key indicator of neurodevelopmental disruptions. However, in our study, walking was not found to be associated with PE. Instead, balance and ball handling—skills that require coordination, postural control, and more refined motor integration—emerged as the most predictive motor indicators of adolescent PE. This suggests that more complex motor tasks, involving greater cortical–subcortical integration, may be more closely linked to psychosis risk than earlier milestones like walking.

A second possible explanation might be found in the difficulties of accurately assessing delusions (Staines et al. 2022). Delusions might be determined to be part of (sub)culturally normo‐conforming religion, spirituality, or other belief systems more than hallucinations (Coughlan et al. 2022). It is difficult to assess whether the demarcation of various beliefs as either delusion or fitting within a certain social context is justified.

This study has several strengths, the first being that the study sample is from a large population‐based enriched cohort of high‐risk adolescents based on their self‐reported emotional and behavioral problems. Second, a detailed questionnaire was used to measure PE, which allowed for separate analyses of both hallucinatory and delusional experiences, offering deep phenotypic insights and detailed information on comorbid problems.

Several limitations of this study should be considered when interpreting the results. First, childhood impaired motor development was reported by parents retrospectively when adolescents were 15 years old; it is difficult to estimate whether this could lead to over‐ or underreporting of impaired motor development, but the skewed distribution towards “above average” scores indicates a possibility of overestimation of the child's development and therefore a larger possibility of underreporting of impaired motor development. Also, research shows that recall bias favors underreporting as time increases, especially given the transient nature of the observed phenomena (Walter 1990). Although the categorization of our data limits direct comparison, when we compare our data for onset of walking with the Bayley‐III‐NL, the results are consistent (Van Baar AL et al. 2015). The median age of 15 months reported in the Bayley‐III‐NL aligns with our 13–17 months category. In addition, 92% of our adolescents were reported to have walked at or before 17 months, closely matching the 90th percentile of Bayley for 17 months for this milestone, which argues against significant under‐ or overreporting. Second, similarly to existing research on this topic, the questionnaire used to assess impaired motor development was not specifically compiled for assessing motor development or function but rather selectively taken from other validated instruments, allowing for a broad evaluation within a large cohort. Consequently, this approach may introduce inaccuracies in capturing impaired motor development with high precision. Furthermore, our data did not allow for a clear distinction between developmental motor delays and other impaired motor functions such as motor retardation or agitation, which have been found to be differently associated with psychopathology in different ways (Burton et al. 2023; Walther and Mittal 2022). Future studies using comprehensive clinical‐rating scales could further refine these directions. Similarly, an interesting addition would have been to assess motor abnormalities at the time of measuring PE, allowing for further analysis of interaction between motor development stages and motor abnormalities, potentially establishing additive vulnerability, as demonstrated in a recent publication by Fattal et al. (2024). Finally, because data were more complete in adolescents from higher socioeconomic families, we cannot rule out that selective nonresponse bias influenced our findings.

A major recommendation for further research is the development of more accurate rating instruments for the assessment of motor abnormalities and signs of impaired motor development. A second recommendation is the longitudinal study of the development of psychosis spectrum disorders from subclinical symptoms. Such studies would facilitate deeper exploration into the diagnostic relevance and predictive value of both impaired motor development and PE, including their potential as transdiagnostic markers.

## 5 Conclusion

To conclude, this study showed that adolescents with childhood impaired motor development are more likely to have PE, specifically hallucinatory experiences. This underlines that childhood impaired motor development could be a potential early marker for psychosis spectrum disorders. Furthermore, this study substantiates the understanding of childhood impaired motor development as a sign of risk of psychopathology and might thus be recognized through other expressions of altered neurodevelopment.

## Ethics Statement

This study was conducted in accordance with the guidelines of the World Medical Associations Declaration of Helsinki and was approved by the Medical Ethical Review Committee of the Erasmus MC University Medical Center, Rotterdam. Procedures were fully explained to the participants.

## Consent

Both adolescents and their parent(s) or legal guardians gave written informed consent to participate in the study.

## Conflicts of Interest

The authors declare no conflicts of interest.

## Supporting information




**Supplementary Table 1**. Non‐response analysis for adolescents who had available data for impaired motor development (responders) compared to those who were missing the data (non‐responders)
**Supplementary Table 2**. Sensitivity analyses of the associations between childhood impaired motor development and adolescent psychotic experiences, adjusted for emotional and behavioral problems

## Data Availability

Data materials and coding that support the findings of this study are available upon request to the principal investigator of the iBerry study.
