# Supplementary material for: The Association Between Childhood Impaired Motor Development and Adolescent Psychotic Experiences
Source: Dev Psychobiol. 2025 May 14;67(3):e70049. doi: 10.1002/dev.70049 (PMC12078880; doi:10.1002/dev.70049)
Supplement: Supplementary file 1 — Supplementary Table 1. Non‐response analysis for adolescents who had available data for impaired motor development (responders) compared to those who were missing the data (non‐responders) Supplementary Table 2. Sensitivity analyses of the associations between childhood impaired motor development and adolescent psychotic experiences, adjusted for emotional and behavioral problems [file DEV-67-e70049-s001.docx]

| **Supplementary Table 1** Non-response analysis for adolescents who had available data for impaired motor development (responders) compared to those who were missing the data (non-responders) | | | | | | | |
| --- | --- | --- | --- | --- | --- | --- | --- |
| **Variable** | **T0** | | | | **T1** | | |
|  | **Responders**  **(n=661)** | **Non-responders**  **(n=361)** | ***p*-value^a^** | **Responders**  **(n=590)** | | **Non-responders**  **(n=217)** | ***p*-value^a^** |
| Age, mean (SD) | 14.7 (0.76) | 15.5 (0.99) | 0.098 | 17.9 (0.76) | | 18.6 (0.89) | 0.342 |
| Sex, n (%)  Male  Female | 318 (48.1)  343 (51.9) | 182 (50.4)  179 (49.6) | 0.481 |  | |  |  |
| Education level, n (%)  Low  Medium  High  Not specified | 293 (44.7)  152 (23.2)  153 (23.4)  57 (8.7) | 174 (57.0)  67 (22.0)  33 (10.8)  31 (10.2) | < 0.001 | 287 (49.7)  167 (28.9)  124 (21.4) | | 130 (62.2)  44 (21.1)  35 (16.7) | 0.008 |
| Urbanicity  Rural  Suburban  Urban | 138 (20.9)  135 (20.4)  388 (58.7) | 71 (19.7)  57 (15.8)  233 (64.5) | 0.126 | 130 (22.1)  110 (18.6)  350 (59.3) | | 44 (20.3)  31 (14.3)  142 (65.4) | 0.001 |
| Household monthly income, n (%)  < 1600 euro  1600 – 2399 euro  2400 – 4400 euro  > 4400 euro | 52 (8.2)  97 (15.4)  332 (52.5)  151 (23.9) | 56 (23.2)  41 (17.0)  102 (42.3)  42 (17.5) | < 0.001 | 26 (4.4)  77 (13.2)  265 (45.4)  216 (37.0) | | 30 (17.2)  27 (15.4)  66 (37.7)  52 (29.7) | < 0.001 |
| Emotional and behavioral problems, score, median (IQR) | 38 (24) | 44 (29) | 0.075 | 41 (30) | | 44 (33) | 0.149 |
| Total psychotic experience, score, median (IQR) | 2 (4) | 3 (4) | 0.322 | 2 (3) | | 3 (4) | 0.486 |
| SD = Standard Deviation; IQR = Interquartile Range; ^a^ Pearson’s chi-squared test. | | | | | | | |

| Supplementary Table 2 Sensitivity analyses of the associations between childhood impaired motor development and adolescent psychotic experiences | | | | | | | |
| --- | --- | --- | --- | --- | --- | --- | --- |
| Impaired motor development | **Psychotic experiences** | | | | | | |
|  | T0 ­­­̶ mean age = 15.0 years – n = 658 | | | | | | |
|  | Total psychotic experiences | | Hallucinatory experiences | | Delusional experiences | | |
|  | *β* (95% CI) | *p*-value | *β* (95% CI) | *p*-value | *β* (95% CI) | *p*-value | |
| Onset of walking | 0.16 (-0.06;0.37) | 0.15 | 0.09 (-0.12;0.30) | 0.40 | 0.10 (-0.07;0.27) | 0.24 | |
| Aptitude in ballgames | -0.08 (-0.22;0.05) | 0.23 | -0.09 (-0.22;0.04) | 0.18 | -0.07 (-0.18;0.03) | 0.18 | |
| Balance | 0.04 (-0.12;0.21) | 0.60 | 0.09 (-0.07;0.25) | 0.25 | -0.05 (-0.18;0.08) | 0.49 | |
| Smoothness of movement | -0.02 (-0.19;0.16) | 0.87 | 0.03 (-0.13;0.20) | 0.70 | -0.09 (-0.23;0.04) | 0.18 | |
| Sum scores: |  |  |  |  |  |  | |
| Continuous | -0.01 (-0.07;0.05) | 0.80 | -0.00 (-0.06;0.06) | 0.98 | -0.03 (-0.07;0.02) | 0.29 | |
| Above cutoff (≥1 impairment) | 0.00 (-0.12;0.13) | 0.95 | -0.03 (-0.14;0.09) | 0.67 | -0.03 (-0.13;0.06) | 0.48 | |
|  | T1 ­­­̶ mean age = 18.1 years – n = 582 | | | | | | |
|  | Total psychotic experiences | | Hallucinatory experiences | | Delusional experiences | | |
|  | *β* (95% CI) | *p*-value | *β* (95% CI) | *p*-value | *β* (95% CI) | *p*-value | |
| Onset of walking | 0.01 (-0.19;0.21) | 0.91 | -0.04 (-0.23;0.16) | 0.72 | 0.08 (-0.09;0.25) | 0.35 | |
| Aptitude in ballgames | 0.07 (-0.06;0.19) | 0.29 | **0.16 (0.03;0.28)** | **0.01** | -0.08 (-0.19;0.03) | 0.17 | |
| Balance | 0.10 (-0.06;0.25) | 0.22 | **0.25 (0.09;0.40)** | **0.002** | -0.09 (-0.22;0.05) | 0.22 | |
| Smoothness of movement | 0.03 (-0.14;0.19) | 0.76 | 0.13 (-0.04;0.29) | 0.13 | -0.11 (-0.25;0.042 | 0.15 | |
| Sum scores: |  |  |  |  |  |  |  |
| Continuous | 0.03 (-0.03;0.08) | 0.23 | **0.07 (0.02;0.13)** | **0.01** | -0.03 (-0.08;0.02) | 0.21 | |
| Above cutoff (≥1 impairment) | 0.10 (-0.01;0.22) | 0.07 | **0.16 (0.05;0.28)** | **0.004** | -0.00 (-0.10;0.08) | 0.95 | |
|  | Difference between T0 and T1 – n = 580 | | | | | | |
|  | Total psychotic experiences | | Hallucinatory experiences | | Delusional experiences | | |
|  | *β* (95% CI) | *p*-value | *β* (95% CI) | *p*-value | *β* (95% CI) | *p*-value | |
| Onset of walking | -0.08 (-0.79;0.64) | 0.83 | -0.12 (-0.60;0.36) | 0.63 | 0.10 (-0.23;0.43) | 0.54 | |
| Aptitude in ballgames | **0.51 (0.06;0.96)** | **0.03** | **0.47 (0.17;0.77)** | **0.002** | -0.00 (-0.21;0.21) | 0.98 | |
| Balance | 0.26 (-0.31;0.83) | 0.37 | 0.27 (-0.12;0.65) | 0.17 | -0.07 (-0.33;0.19) | 0.60 | |
| Smoothness of movement | 0.23 (-0.37;0.84) | 0.45 | 0.19 (-0.21;0.60) | 0.35 | -0.01 (-0.29;0.27) | 0.94 | |
| Sum scores: |  |  |  |  |  |  | |
| Continuous | 0.17 (-0.03;0.38) | 0.09 | **0.15 (0.02;0.29)** | **0.03** | -0.00 (-0.09;0.09) | 0.99 | |
| Above cutoff (≥1 impairment) | 0.38 (-0.03;0.80) | 0.07 | **0.29 (0.02;0.57)** | **0.04** | 0.09 (-0.10;0.28) | 0.38 | |
| Results from linear regression models, adjusted for age and sex and emotional and behavioral problems; psychotic experiences are assessed with the Prodromal Questionnaire 16; emotional and behavioral problems are assessed with the Youth Self Report total problem scale; | | | | | | | |
